# miR-146a modulates TLR1/2 and 4 induced inflammation and links it with proliferation and lipid production via the indirect regulation of GNG7 in human SZ95 sebocytes

**DOI:** 10.1038/s41598-021-00907-1

**Published:** 2021-11-02

**Authors:** Katalin Dull, Fruzsina Fazekas, Dávid Deák, Dóra Kovács, Szilárd Póliska, Andrea Szegedi, Christos C. Zouboulis, Dániel Törőcsik

**Affiliations:** 1 Department of Dermatology, Faculty of Medicine, University of Debrecen, Nagyerdei krt. 98, Debrecen, 4032 Hungary; 2 Department of Biochemistry and Molecular Biology, Genomic Medicine and Bioinformatics Core Facility, Faculty of Medicine, University of Debrecen, Debrecen, Hungary; 3 Division of Dermatological Allergology, Department of Dermatology, Faculty of Medicine, University of Debrecen, Debrecen, Hungary; 4 Departments of Dermatology, Venereology, Allergology and Immunology, Dessau Medical Center, Brandenburg Medical School Theodor Fontane and Faculty of Health Sciences Brandenburg, Dessau, Germany

**Keywords:** Cell biology, Medical research

## Abstract

Activation of Toll-like receptors (TLR) 1/2 and 4 are central in inducing inflammation in sebocytes by regulating the expression of protein coding mRNAs, however the microRNA (miRNA) profile in response to TLR activation and thus the possible role of miRNAs in modulating sebocyte functions has not been elucidated. In this work we identified miR-146a to have the highest induction in the TLR1/2 and 4 activated SZ95 sebocytes and found that its increased levels led to the down-regulation of IL-8 secretion, decreased the chemoattractant potential and stimulated the proliferation of sebocytes. Assessing the gene expression profile of SZ95 sebocytes treated with a miR-146a inhibitor, the induction of *GNG7* was one of the highest, while when cells were treated with a miR-146a mimic, the expression of *GNG7* was down-regulated. These findings correlated with our in situ hybridization results, that compared with control, miR-146a showed an increased, while *GNG7* a decreased expression in sebaceous glands of acne samples. Further studies revealed, that when inhibiting the levels of *GNG7* in SZ95 sebocytes, cells increased their lipid content and decreased their proliferation. Our findings suggest, that miR-146a could be a potential player in acne pathogenesis by regulating inflammation, inducing proliferation and, through the indirect down-regulation of *GNG7*, promoting the lipid production of sebocytes.

## Introduction

Toll-like receptors (TLRs) belong to the family of pattern-recognition receptors and are key players in the innate immune system to selectively sense the presence of various microorganisms^1–3^. Importantly, elevated expression of TLR2 and TLR4 were reported in acne-involved skin^4,5^, suggesting that these pathways may also be involved in acne pathogenesis. While TLR2 recognizes peptidoglycans, lipoproteins, lipoarabinomannans and short-chain fatty acids from Gram-positive bacteria, TLR4 is activated by the Gram-negative bacterial component lipopolysaccharide (LPS)^6^. Interestingly lipids, such as saturated fatty acids, are also able to activate both TLR2 and TLR4^7–9^. Indeed, products of *Cutibacterium acnes (C. acnes),* a Gram-positive, anaerobic bacterium, which behaves both as commensal and pathogen in acne skin, and several sebaceous lipids, among them palmitic acid, whose altered ratios were detected in acne patients, are possible TLR activators in acne lesions^10–17^.

Sebaceous glands are known for their primary role to secrete and metabolize lipids leading to the production of sebum to moisturize the hair and the skin, which feature is regulated by a wide repertoire of stimuli such as hormones, lipids and pathogens both in physiological as well as in disease settings^18^. However, with a great number of proteins and lipids that exert inflammatory properties, sebocytes are also actively involved in shaping the inflammatory environment^19–21^, in which their activation through TLRs^14,22,23^ might play a central role. Supporting this pustulate, our previous genome wide gene expression study showed that sebocytes are able to rapidly gain and prioritize an immune-competent status in response to TLR1/2 and/or TLR4 activation at the level of mRNA expression^22^.

MicroRNAs (miRNA) are small non-coding RNAs also selectively transcribed from the genome under various conditions. In contrast to mRNAs they do not encode proteins but control gene expression by binding and destabilizing their target mRNA^24^. Therefore, they regulate transcriptional and post-transcriptional gene expression. In human sebocytes, by transfecting SZ95 sebocytes with siRNAs directed against DICER, a key enzyme of miRNA biogenesis, miRNA presence was confirmed and was proven essential for lipogenesis, but without providing any disease-specific conclusions on their possible role^25^. Moreover using whole tissue samples, miRNAs were also linked to sebaceous gland associated tumors^26–29^, however the cellular source of the differentially expressed miRNAs was not assessed.

In the present study, we aimed to extend our knowledge on the gene expression regulation and profile of TLR-activated sebocytes and investigated the role of miRNAs in it. We found that in TLR1/2- and 4-activated sebocytes, miRNAs had altered expression levels, with miR-146a showing the most prominent upregulation. Confirming that sebaceous glands of acne samples also exhibited high expression levels of miR-146a, we aimed to define a possible pathophysiological role for miR-146a in sebocytes. Our results suggest that miR-146a may not only regulate TLR-induced inflammation in sebocytes but could be a missing link in connecting it with hyperproliferation and increased sebum production by indirectly regulating the expression of G protein gamma 7 (*GNG7*), which may have both pathological and therapeutic implications in sebaceous gland-associated diseases, such as acne.

## Results

### miR-146a shows the most prominent induction in TLR1/2 and 4-stimulated SZ95 sebocytes

**Figure 1:**
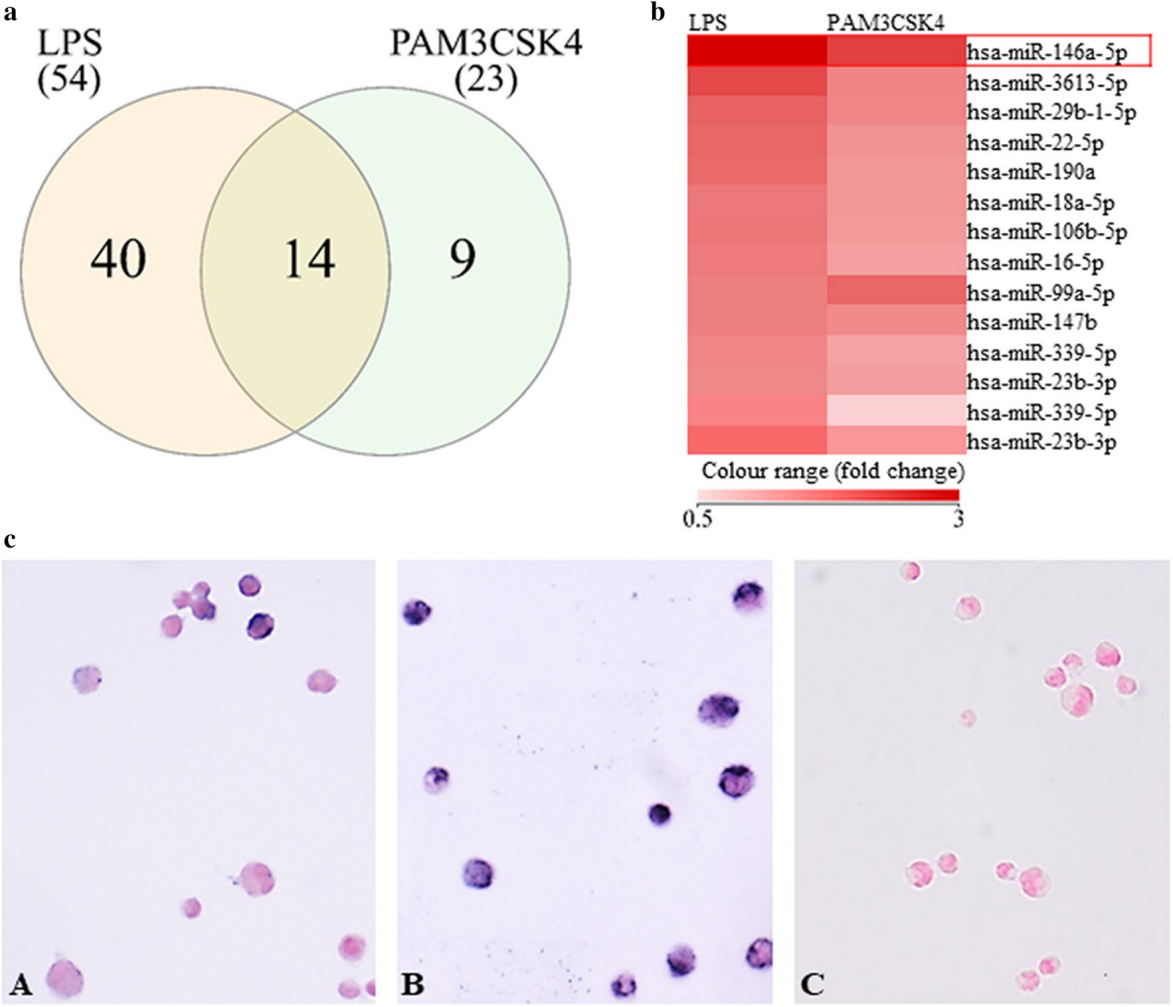
(**a**) Venn diagram showing the numbers of the upregulated miRNAs in TLR1/2 and TLR4 activated SZ95 sebocytes. 14 miRNAs were upregulated 24 h after both LPS and PAM3CSK4 treatments as revealed by our RNAseq measurements. (**b**) Heatmap visualizing the 14 miRNAs’ fold-change values after TLR activation. miR-146a showed the most significant upregulation in both treatments. (**c**) Detection of miR-146a by in situ hybridization in SZ95 sebocytes. Compared to untreated SZ95 sebocytes (**A**), a more intense blue cytoplasmic hybridization signal was detected in TLR1/2 activated (24 h PAM3CSK4 treatment) sebocytes (**B**); negative control staining (**C**) as described in “Methods”. Chromogenic in situ hybridization, NBT/BCIP blue chromogen reaction with nuclear Fast Red background staining. Original magnification × 200. Experiments were carried out in biological triplicates.

Sebocytes are able to sense and respond to different TLR stimuli^22^, making them an active player in a pathogen-associated inflammatory environment. To investigate the change in their miRNA profile in such a response, we applied two different TLR activators, PAM3CSK4 (TLR1/2 activator) and LPS (TLR4 activator), to treat human SZ95 sebocytes. Performing genome wide expression studies in samples treated for 24 h, 23 miRNAs responded to PAM3CSK treatment, while 54 miRNAs were significantly upregulated after LPS treatment (Supplementary Table 1A-B). Both TLR activators induced common significant elevation of 14 miRNA expression levels (Fig. 1a), of which miR-146a showed the most abundant values (Fig. 1b). This significant increase in the levels of miR-146a could also be detected by in situ hybridization in TLR1/2- and 4-activated SZ95 sebocytes (Fig. 1c).

### miR-146a is elevated in sebaceous glands of human acne tissue samples

**Figure 2:**
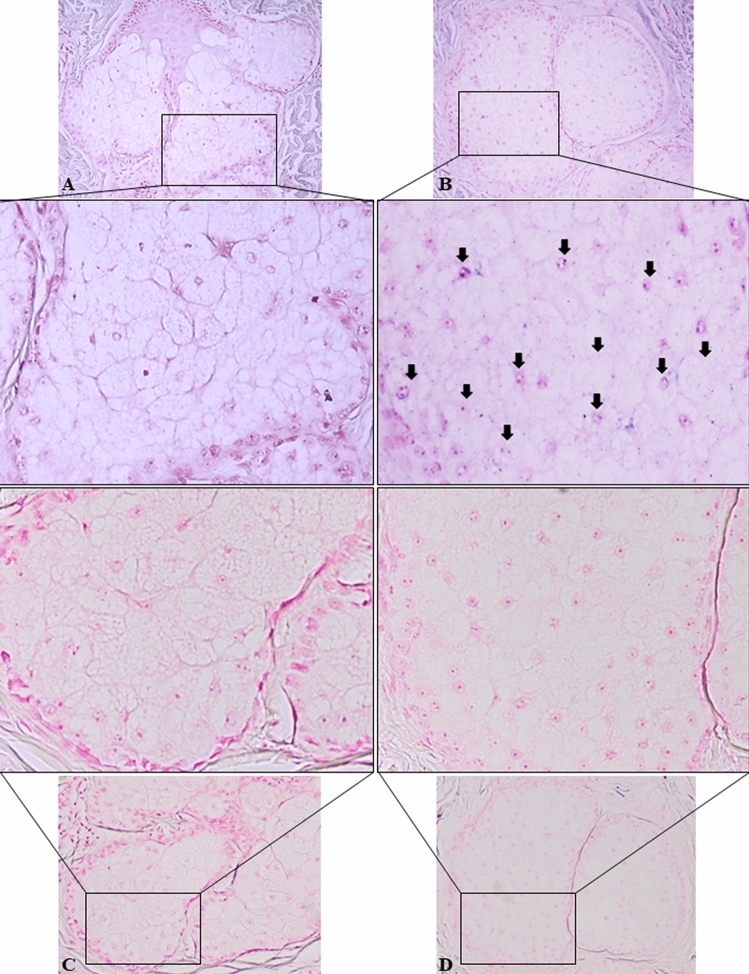
In situ detection of miR-146a on FFPE tissue samples. Compared with normal skin samples (**A**), a more intense, granular blue hybridization signal was detected in sebaceous glands of acne vulgaris samples (**B**). Negative control staining of normal (**C**) and acne vulgaris (**D**) skin samples as described in “Methods”. Samples obtained from the back of patients. Arrows show miR-146a positive sebocytes. Chromogenic in situ hybridization, NBT/BCIP blue chromogen with nuclear Fast Red background staining. Original magnification × 200, Representative microphotograph, n = 5.

To provide a biological relevance for our finding, we performed in situ hybridization against miR-146a in 5 acne vulgaris and 5 normal skin samples from the back of young male adults. While in normal skin, miR-146a was detectable with low-intensity homogeneous staining, a more intense, mainly granular staining was observed in sebaceous glands of acne tissue samples (Fig. 2).

### miR-146a decreases IL-8 secretion and negatively regulates the chemoattractant potential of SZ95 sebocytes

To assess the possible role of miR-146a, we focused on the functional analysis of SZ95 sebocytes transfected with hsa-miR-146a mimic, hsa-miR-146a inhibitor, or their negative controls for 72 h.

**Figure 3:**
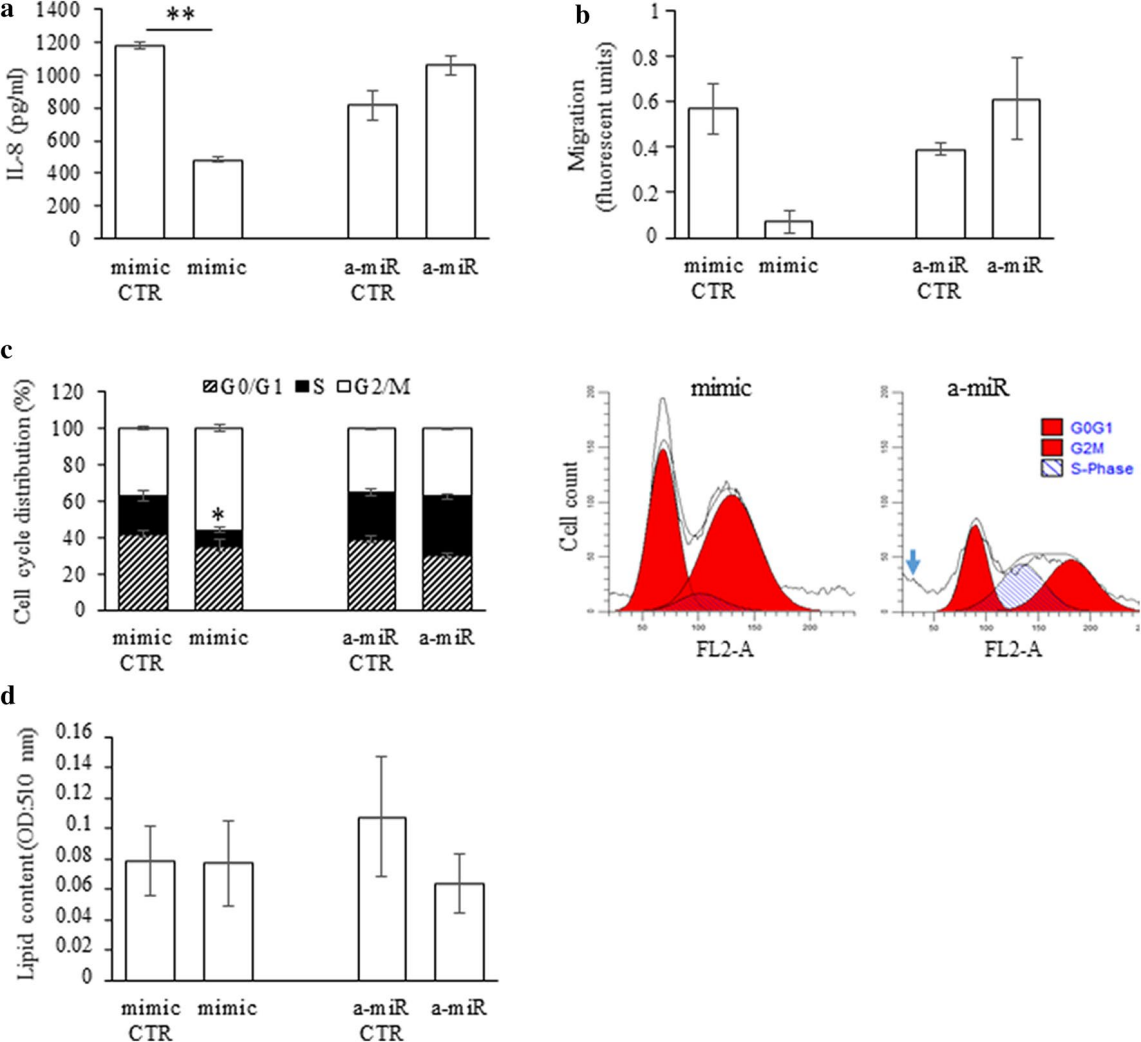
Characterization of the effects of miR-146a on sebocyte functions. SZ95 sebocytes were transfected with miR-146a mimic- (mimic), miR-146a inhibitor (a-miR) or their relevant negative control (mimic CTR for mimic; a-miR CTR for inhibitor) sequences as described in “Methods”. (**a**) Transfection with miR-146a mimic caused a decrease (481.3 ± 32.7 versus control 1178.4 ± 57.2 pg/ml, p-value: 0.002), while with miR-146a inhibitor caused an increase (1056.1 ± 36 versus 813.5 ± 55.5 pg/ml) in the secreted IL-8 levels when compared to transfection with control sequences as measured from the supernatants of SZ95 sebocytes by ELISA. (**b**) Migration assay revealed that monocytes migrated less towards mimic and more towards inhibitor transfected sebocyte supernatants when compared to negative controls. Graphs show the fluorescence intensity of migrated cells as described in “Methods”. (**c**) Measurement of G0, G1, S, G2, M cell cycle phases and apoptosis in SZ95 sebocytes treated with miR-146a mimic, miR-146a inhibitor (a-miR) or their negative controls (mimic CTR for mimic; a-miR CTR for inhibitor) for 72 h. Graphs show the percentage of cells in each phase based on the DNA content analysis measured by flow cytometer. Cell populations treated with mimic sequence showed a significantly lower number of cells in S phase (8.69% versus 21.51% of cells, p-value: 0.05) and larger in G2/M phase (56.02% versus 36.78% of the cells) compared to samples treated with negative control sequences. In the sebocyte cultures treated with inhibitor, more cells were found in S phase (32.62% versus control 25.95% of cells) and less in G0/G1 phase (30% versus 38.93%) when compared to samples treated with negative control sequences. Note the sub-G0/G1 peak in the histogram, representing apoptotic cells (blue arrow). Total cell count: 20.000 for each sample. (**d**) Oil Red O staining of SZ95 sebocytes. Treatment with miR-146a mimic or with miR-146a inhibitor sequence caused no significant change in the lipid content. Graphs show the mean optical density of samples. FU: fluorescence unit. All experiments were carried out in biological triplicates. P-values were calculated using non-parametric Mann–Whitney test, p-values < 0.05 were considered as statistically significant.

First, we measured the secreted interleukin 8 (IL-8) levels from supernatants, which are known to be negatively regulated by miR-146a^30^, 72 h after transfection by ELISA. Cells transfected with hsa-miR-146a mimic sequence secreted significantly lower amounts of IL-8 (481.3 ± 32.7 versus control 1178.4 ± 57.2 pg/ml, p-value: 0.002), while the inhibition of miR-146a resulted in elevated IL-8 secretion (1056.1 ± 36 versus 813.5 ± 55.5 pg/ml) (Fig. 3a), confirming its negative regulatory role in the inflammatory response also of sebocytes.

As IL-8 is an important chemokine in immune cell migration, we examined the migratory capacity of peripheral blood monocytes towards supernatants of transfected SZ95 sebocytes. We observed a lower migration to hsa-miR-146a mimic-transfected and an increased migration towards hsa-miR-146a inhibitor treated SZ95 sebocyte supernatants, showing that miR-146a may have a negative regulatory role on monocyte chemoattraction under inflammatory conditions (Fig. 3b).

### miR-146a regulates cell proliferation of SZ95 sebocytes

To examine the role of miR-146a in sebocyte proliferation and lipid production of cells treated with hsa-miR-146a mimic, hsa-miR-146a inhibitor, or their negative controls for 72 h, we analyzed the cell numbers in different cell cycle phases using DNA content histograms and applied Oil Red O staining to detect lipids.

When incubated with miR-146a mimic sequence, the proportion of cells in G2 and M phases increased (56.02% versus 36.78% of the cells) with a significant decrease in S phase (8.69% versus 21.51% of cells, p < 0.05) and a slight decrease in G0 and G1 phases (35.28% versus 41.71% of cells) in comparison with control (Fig. 3c), showing that cell proliferation is stimulated by higher miR-146a levels. Transfecting SZ95 sebocytes with miR-146a inhibitor, the cell proportion in the S phase increased (32.62% versus control 25.95% of cells) and the G0/G1 population decreased (30% versus 38.93%), suggesting that the mitotic machinery itself was activated but might be blocked in S phase. According to the DNA histogram, a peak of apoptotic cells was also observed following miR-146a inhibitor treatment.

Lipid measurements found a slight, but statistically not significant decrease in the lipid content of miR-146a inhibitor-treated cells and no change in cells treated with the mimic sequence (Fig. 3d).

### Whole transcriptome analysis with clustering revealed changes in genes related to inflammation and cell proliferation in miR-146a inhibitor-treated SZ95 sebocytes

**Figure 4:**
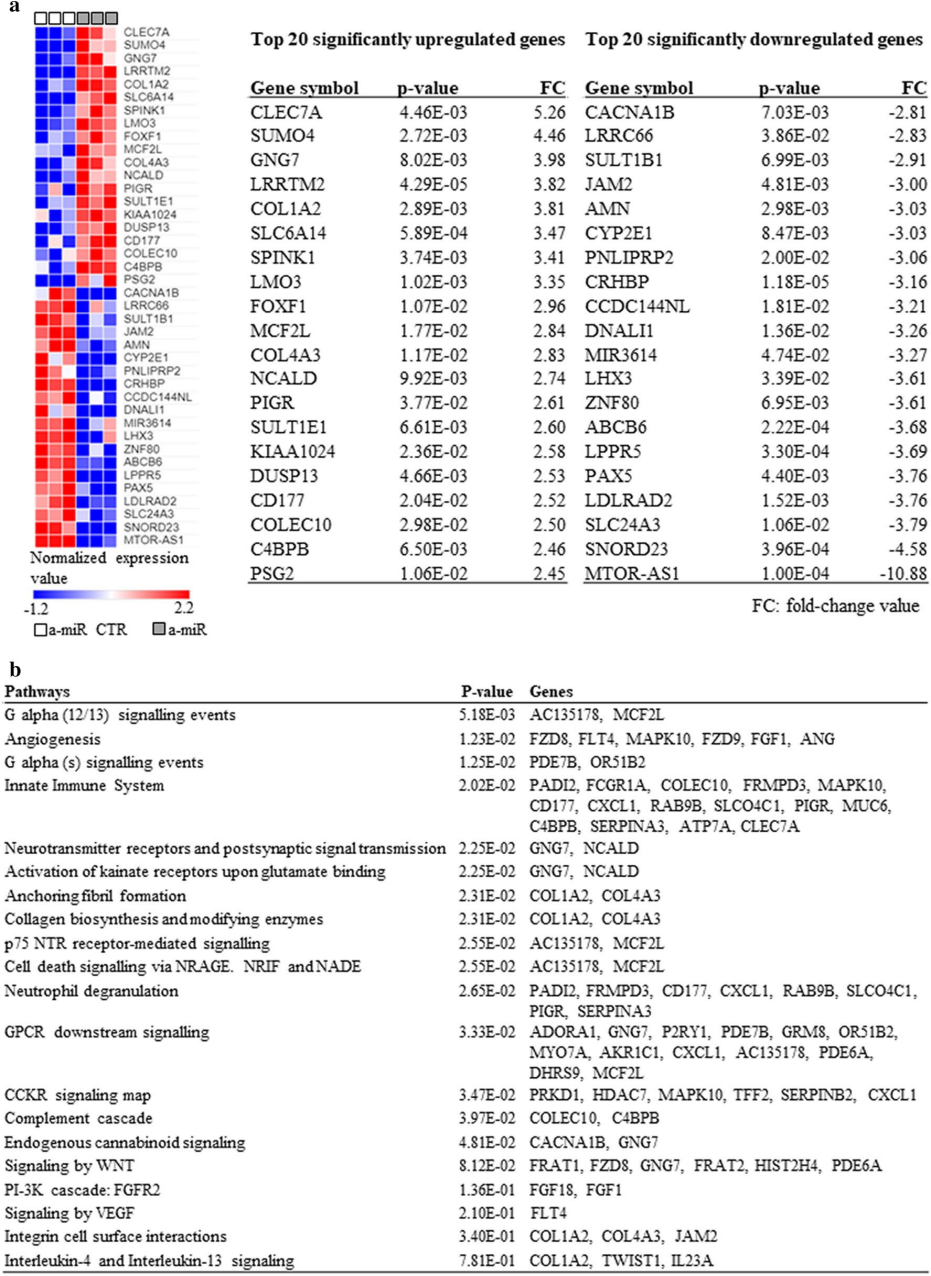
Whole transcriptome analysis of SZ95 sebocytes treated with miR-146a inhibitor for 72 h (n = 3). (**a**) Heatmap visualization and the normalized expression values of the top twenty significantly up- and downregulated genes as revealed by our RNAseq measurements. P-values were calculated using moderated *t* test method, p-values < 0.05 were considered as statistically significant. (**b**) Pathway analysis of significantly regulated genes using PANTHER Classification System (v.16.0). P-values were calculated using Mann–Whitney U test (integrated to PANTHER Classification System), p-values < 0.05 were considered as statistically significant.

To detect how levels of miR-146a could impact changes at the level of gene expression in sebocytes, we performed whole transcriptome analysis of miR-146a inhibitor-treated cells at 72 h and found that 157 genes were significantly upregulated, while 145 genes were downregulated (Supplementary table 2A-B). The top 20 up-, and the top 20 downregulated genes, based on the changes in their expression levels, are shown in Fig. 4a. Functional gene clustering confirmed that the altered levels of miR-146a in sebocytes may lead to changes in immune response, angiogenesis and signaling pathways such as Wnt or G protein mediated ones (Fig. 4b).

### miR-146a levels in sebocytes lead to changes in the expression of GNG7

**Figure 5:**
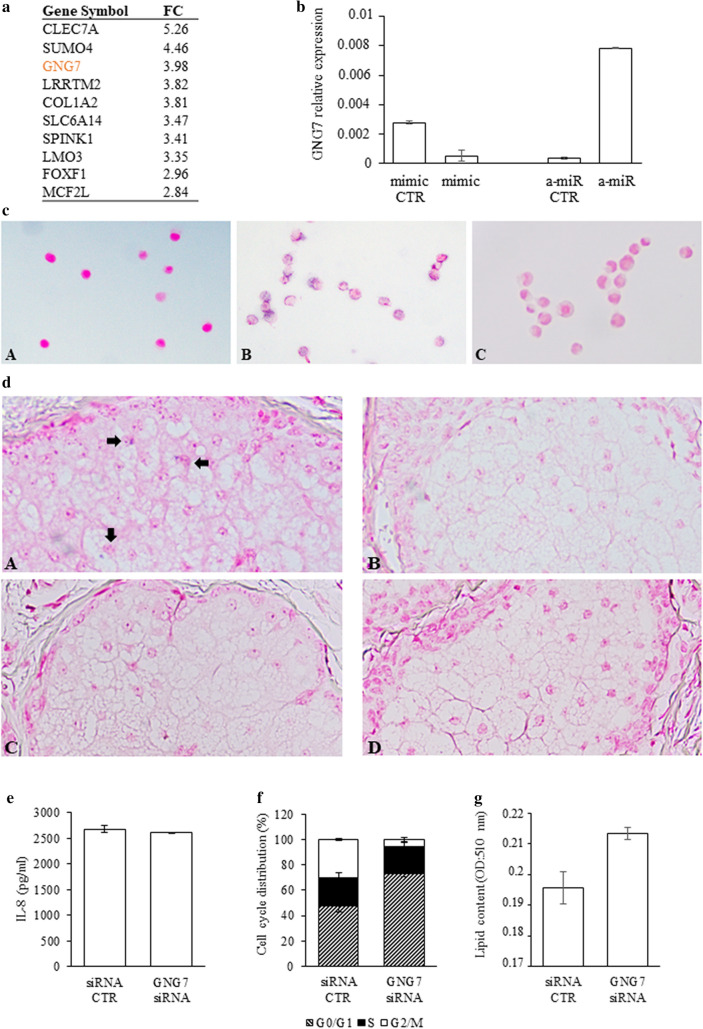
Detection and characterization of the effects of GNG7 on sebocyte functions. (**a**) Genes with the highest fold-change (FC) values in their expression levels in miR-146a inhibitor treated SZ95 sebocytes as revealed by our RNAseq measurements. (**b**) Changes of relative *GNG7* mRNA levels measured by qRT-PCR in SZ95 sebocytes normalized to *PPIA*. Note that *GNG7* mRNA levels decreased in SZ95 sebocytes transfected with miR-146a mimic (mimic) and increased in the inhibitor treated cells (a-miR) when compared to their control sequences (mimic CTR and a-miR CTR respectively). (**c**) in situ hybridization for the detection of *GNG7* mRNA in SZ95 sebocytes. Note, that when compared to miR-146a mimic (**A**), a more intense blue hybridization signal was seen in the cytoplasm of the miR-146a inhibitor treated SZ95 sebocytes (**B**). (**C**) Negative control staining. (**d**) in situ hybridization for the detection of *GNG7* in sebaceous glands of normal (**A**) and acne vulgaris (**B**) FFPE human tissue samples. Note that while in acne samples, *GNG7* mRNA could not be detected, hybridization signals were observed in healthy skin samples. Arrows show *GNG7* mRNA-positive sebocytes. Negative control staining of normal (**C**) and of acne vulgaris samples (**D**) as described in “Methods”. Representative photomicrograph, n = 5. Chromogenic in situ hybridization, NBT/BCIP blue chromogen with nuclear Fast Red background staining. Original magnification × 400. (**e**) Silencing of *GNG7* mRNA does not affect IL-8 production (**e**), but promotes SZ95 sebocyte differentiation according to our cell cycle analysis (**f**) and lipid content measurements (**g**).

According to the transcriptome analysis, *GNG7* had one of the highest upregulation following miR-146a inhibition (Fig. 5a). The miR-146a mediated expression changes of *GNG7* could be further confirmed by qRT-PCR and in situ hybridization in SZ95 sebocyte cultures transfected with mimic, inhibitor and control sequences (Fig. 5b,c), suggesting a central role for *GNG7* in miR-146a-induced signaling.

To assess if *GNG7* mRNA could be also detected in vivo, in situ hybridization was performed on human acne and normal skin samples. The presence of *GNG7* mRNA could be visualized in sebaceous glands of normal skin, while in acne samples, in which elevated miR-146a levels were shown, it could not be detected (Fig. 5d).

### Decreased levels of GNG7 promote differentiation in SZ95 sebocytes

To reveal the roles of *GNG7* in sebocytes, next we performed RNA silencing experiments, using siRNA against *GNG7* mRNA. According to our findings, down-regulation of *GNG7* in SZ95 sebocytes did not affect IL-8 secretion (Fig. 5e) but caused notable changes in cell proliferation (Fig. 5f). The cell population transfected with siRNA, showed elevated proportion of G0/G1 cells (73% vs control 47%, p-value: 0.1) and decreased proportion of G2/M cells (5% vs control 30%, p-value: 0.1) compared to cell populations transfected with negative control sequences showing that the mitotic cascade is less active, the majority of the cells are not proliferating. Performing lipid measurements, we found a tendentious elevation in the lipid content of *GNG7* siRNA-treated SZ95 sebocytes compared to negative control ones (p-value: 0.15, Fig. 5g).

## Discussion

The application of a system-based approach of whole genome sequencing of SZ95 sebocytes, treated with specific and selective TLR1/2 and TLR4 activators, provided evidence that miRNAs are selectively induced in sebocytes upon TLR activation. Moreover, identifying miR-146a as the miRNA with the most abundant induction with an increased expression also in the sebaceous glands of acne samples, our work is the first to identify a miRNA, which is increased in sebaceous glands in a non-malignant, inflammatory disease setting. Characterizing sebocytes with altered levels of miR-146a, we showed that miR-146a is not only a marker for activation, but could have a regulatory role on cell proliferation and on the immune-competence of sebocytes. Our further findings, that *GNG7* was found to be oppositely regulated both in SZ95 sebocytes as well as in sebaceous glands of acne samples, suggests a cascade of events in which the induction of miR-146a leads to proliferation, however the consecutive down-regulation of *GNG7* promotes lipid production of sebocytes.

In line with our previous results, showing that TLR1/2 and TLR4 pathways induced a similar change in the mRNA profile of sebocytes, miRNAs also changed similarly in response to the used activating agents. This finding further supports our previously raised hypothesis that these pathways and the related changes are not stimulus-/pathogen-specific in sebocytes as these receptors can be activated with a wide range of stimuli both of pathogenic and of non-pathogenic origin^22^. In other words, sebocytes use these receptors to sense changes in their environment, such as an altered microbiome or the presence of lipids, which activation needs to be further modulated to gain its disease specific role. Based on our results miR-146a may be a potent regulator, just as it is observed in various cell types of lymphoid, myeloid and of non-immune origin, where miR-146a decreases the production of inflammatory cytokines^31^. Indeed, regarding dermatological diseases, increased levels of miR-146a was already confirmed in keratinocytes of acne just as in psoriasis and atopic dermatitis samples, with a suggested role to regulate inflammation. Moreover, in psoriasis, its genetic alterations even showed an association with disease severity^32–35^. Higher levels of miR-146a was also detected in keratinocytes treated with LTA (TLR2 activator), where it may down-regulate *C. acnes*-induced production of IL-6, -8, and TNF-α by inhibiting the TLR2/IRAK1/TRAF6/NF-κB and MAPK pathways^36^. Our findings that miR-146a was also highly expressed in sebaceous glands of acne samples, confirms that miR-146a may be involved in acne also at the level of sebocytes and adds further important details on the immune-competence of this cell type. Therefore, one of the most interesting findings is that the induction of the TLR-miR-146a axis in sebocytes may result in a decreased production of IL-8, a cytokine characteristic in acne-related inflammation, and in a decreased chemoattractant potential of sebocytes, a feature that was recently reported by our group^19^. Speculating on the in vivo relevance of this finding, it is reasonable to put forward that the increased levels of miR-146a in sebocytes could serve as a negative regulator of inflammation in acne lesions with an impact on the production of inflammatory cytokines and the number of infiltrating immune cells.

Further results showed that the complex changes induced by miR-146a may go beyond changing the inflammatory properties of sebocytes. While the proliferation and apoptosis of sebocytes were dependent on the levels of miR-146a, the increased levels led to an increased proliferation while decreased ones to apoptosis, our unbiased strategy of whole transcriptome analysis performed on sebocytes treated with a specific miR-146a inhibitor, revealed that miR-146a may also influence the gene expression profile of sebocytes. Although the exact mechanisms remain to be elucidated, in inhibitor-treated sebocytes, pathways with pivotal roles in sebocyte functions, such as Wnt or G protein mediated signalling, might be altered at the level of transcription^37^.

**Figure 6:**
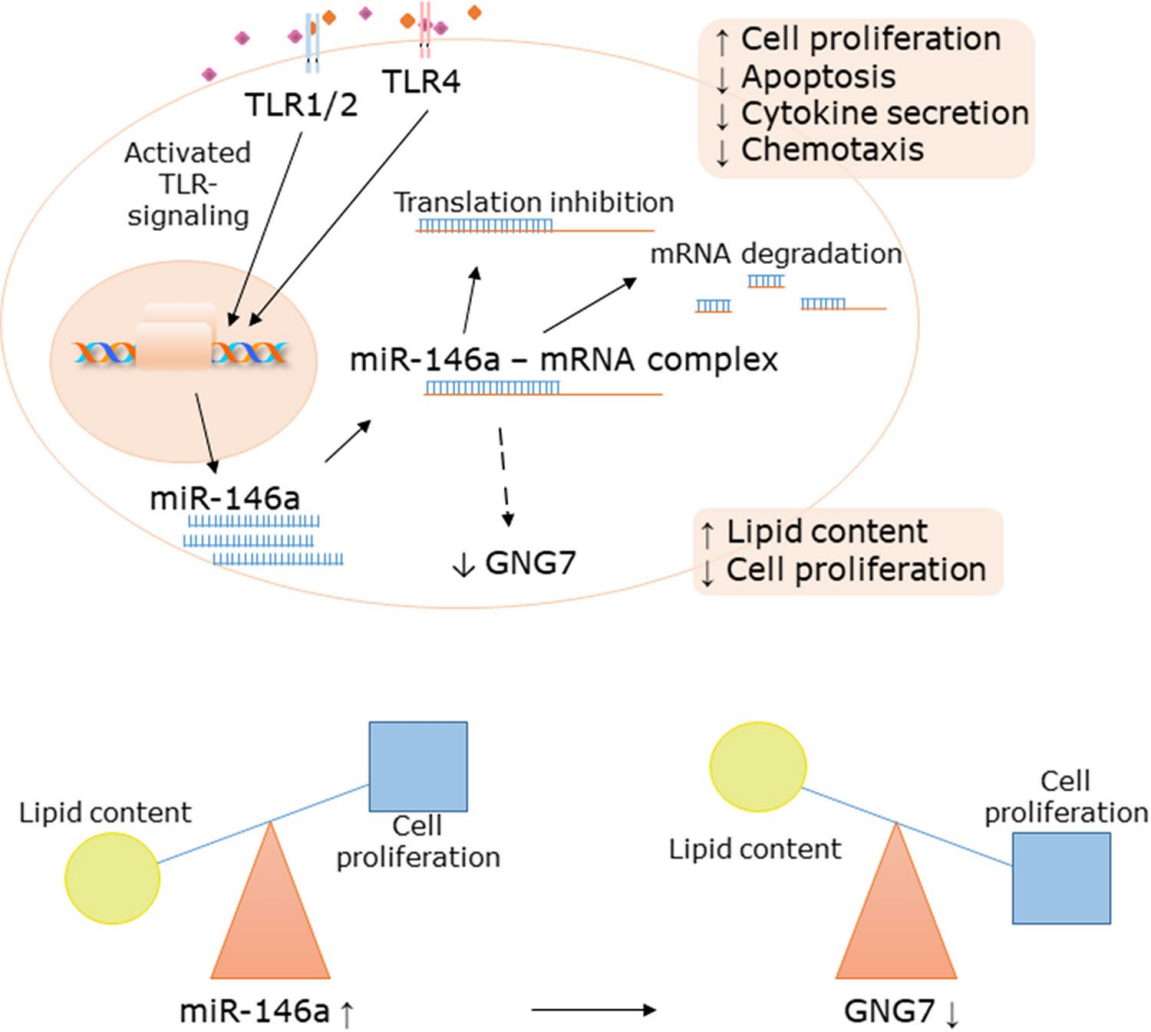
miR-146a links TLR-induced inflammation with cell proliferation and indirectly affects the lipid content of sebocytes via *GNG7*. Our data showed that in response to TLR1/2 and -4 activation the expression of miR-146a, a microRNA leading to the degradation of its target mRNAs, is abundantly induced in sebocytes. The increased levels of miR-146a, observed also in acne samples, may have a negative regulatory effect on cytokine secretion and chemotaxis, induce cell proliferation and decrease apoptosis, while the parallel down-regulation of *GNG7*, may shift the balance from proliferation to lipid accumulation. Figure was created with BioRender.

Based on the fold-change values, the functional clustering of the differentially expressed genes in miR-146 inhibitor treated SZ95 sebocytes and the in situ hybridization studies, *GNG7* came into the focus showing an opposite regulation with miR-146a both in sebocytes and in sebaceous glands of acne samples, in which the expression of miR-146a increased while *GNG7* was not detectable compared to control skin samples. Our findings revealed, that such decrease in the levels of *GNG7* may promote sebocyte differentiation and as a result to an increased sebum secretion. Interestingly, although *GNG7* has not been detected in the skin yet, in the nervous tissue where it is known to be predominantly expressed^38^, *GNG7* inhibited cell proliferation, promoted cell differentiation and induced cell death by inhibiting mTOR signalling^39,40^. The findings that the activation of mTOR pathway is also involved in the regulation of sebocyte proliferation and maturation, and its induction by various agents is central in the development of acne^41–44^, make *GNG7* an interesting candidate for further studies in sebocyte biology (Fig. 6).

In summary, our findings may bring us closer to understand the morphological and functional changes of sebaceous glands observed in acne, where the characteristic changes of increased sebum production assumes that both the proliferation and the lipid production of sebocytes are stimulated at the same time. Based on a recent publication^45^ reporting that the pathogenetic basis of acne is the alteration in sebocyte differentiation, the less differentiated and with that the more proliferating the sebocytes are the more they are responding to regulatory stimuli, therefore our results point on the therapeutic relevance which the modulation of miR-146a, and with that *GNG7* levels, may deliver to acne therapy by targeting inflammation, sebocyte hyperproliferation and sebum secretion at the same time.

## Methods

### Cells, transfection and treatment

Immortalized human SZ95 sebocytes were maintained as adherent culture at 37 °C in a humidified chamber containing 5% (v/v) CO_2_ in Sebomed basal medium (Sigma-Aldrich, St. Louis, MO, USA) as previously described^46^.

For microRNA profiling, SZ95 cells were treated with 1 μg/ml PAM3CSK4 (TLR1/2 activator; dissolved in sterile water; Cat. no.: TLRL PMS, InvivoGen, San Diego, CA, USA) and 1 μg/ml LPS (TLR4 activator; derived from Escherichia coli; dissolved in sterile water; Cat. no.: L4391, Sigma-Aldrich) for 24 h.

For studies on transfected cells, SZ95 sebocytes were transfected with 25 nM hsa-miR-146a-5p power inhibitor (miRCURY LNA miRNA Power Inhibitor, Cat No: 339131YI04100676-DDA, Qiagen, Hilden, Germany) or negative control (miRCURY LNA miRNA Power Inhibitor Control, Negative Control A, Cat No: 339136 YI00199006-DDA, Qiagen), and 1 nM hsa-miR-146a-5p mimic (mirVana miRNA mimic, Cat. No: 4464066, Ambion, Austin, TX, USA) or negative control (mirVana miRNA mimic Negative Control #1, Cat. No: 4464058, Ambion), 50 nM *GNG7* siRNA (MISSION esiRNA, Cat. No: EHU149141, Sigma-Aldrich) or negative control (MISSION esiRNA universal negative control 1, Cat. No: SIC001, Sigma-Aldrich) using Lipofectamine 2000 (Invitrogen, Carlsbad, CA, USA) at 50,000 cells/well seeding density on 24 well plates. All experiments were carried out in triplicates. Cells and supernatants were harvested at 72 h post-transfection for further analysis.

### Histological samples

Formalin-fixed and paraffin-embedded (FFPE) human skin tissue samples from the Department of Dermatology, University of Debrecen were used after approval of the Regional and Institutional Ethics Committee (Approval ID: UD REC/IEC No. 4103–2014). The study was conducted according to the guidelines of the Declaration of Helsinki. Normal control skin samples were selected from the archive of the Department of Dermatology. Specimens from subjects with papulopustular acne were surgically resected from the back area, fixed in 10% buffered formaldehyde for 24 h, and processed routinely into paraffin blocks. Informed consent was obtained from all subjects involved in the study. Five normal and five acne vulgaris skin samples were evaluated.

### ELISA

Secreted IL-8 protein levels were quantified with IL-8 DuoSet ELISA Kit (R&D Systems, Minneapolis, MS, USA) according to the manufacturer’s instructions. Supernatants were collected after 72 h of transfection, centrifuged and stored at − 20 °C until use. 3,3′,5,5′-Tetramethylbenzidine (TMB, Sigma-Aldrich) was used as visualizing reagent, substrate reaction was stopped with 1 M H_2_SO_4_. Optical density was measured with Epoch microplate spectrophotometer (BioTek, Winooski, VT, USA) at the wavelength of 450 nm.

### RT-qPCR

Total RNA was extracted using TriReagent (Molecular Research Center, Cincinnati, OH, USA) according to the provided protocol. RNA concentration and purity were measured with Nanodrop 2000 (Thermo Scientific, Waltham, MA, USA). Before reverse transcription (RT)-quantitative polymerase chain reaction (qPCR)), total RNA was reverse transcribed using High-Capacity cDNA Reverse Transcription Kit (Applied Biosystems, Foster City, CA, USA) with random primers. Gene expression levels were analyzed using QuantStudio 12 K Flex Real-Time PCR System (Applied Biosystems) using SYBR Green master mix (Roche, Basel, Switzerland) for *GNG7* (forward primer 5’-3’: GACAATGTCAGCCACTAACAACA; reverse primer 5’-3’: CAGTAGCTCATGAGGTCAGACG). Expression levels were normalized against *PPIA* (forward primer 5’-3’: CAGTGCTCAGAGCTCGAAAGT; reverse primer 5’-3’: GTGTTCTTCGACATCACGGC) using the comparative Ct method.

### RNA-Seq

Small RNA-Seq sequencing libraries were generated from 1 µg total RNA using NEBNext Multiplex Small RNA Prep Set for Illumina (1–48) 96 rxn kit (New England BioLabs, Ipswich, MA, USA), according to the manufacturer’s protocol. Fragment size distribution and molarity of libraries were checked on Agilent BioAnalyzer DNA1000 chip (Santa Clara, CA, USA). Then single read 50 bp sequencing run was performed on Illumina NextSeq 500 instrument (San Diego, CA, USA).

To obtain global transcriptome data high throughput mRNA sequencing analysis was performed on Illumina sequencing platform. Total RNA sample quality was checked on Agilent BioAnalyzer using Eukaryotic Total RNA Nano Kit according to the manufacturer’s protocol. Samples with RNA integrity number value > 7 were accepted for library preparation process. RNA-Seq libraries were prepared from total RNA using Ultra II RNA Sample Prep kit (New England BioLabs) according to the manufacturer’s protocol. Briefly, poly-A RNAs were captured by oligo-dT conjugated magnetic beads then the mRNAs were eluted and fragmented at 94 °C for 15 min. First strand cDNA was generated by random priming reverse transcription and after second strand synthesis step double-stranded cDNA was generated. After repairing ends and adapter ligation steps, adapter-ligated fragments were amplified in enrichment polymerase chain reaction and finally, sequencing libraries were generated. The sequencing run was executed on Illumina NextSeq500 instrument using single-end 75 cycle sequencing.

### RNA-Seq data analysis

Raw sequencing data was aligned to human reference genome version GRCh37 using HISAT2 algorithm and BAM files were generated. Downstream analysis was performed using StrandNGS software (version 2.8, build 230243; Strand Life Sciences, Bangalore, India). BAM files were imported into the software, DESeq1 algorithm was used for normalization. Differential expression p-values were calculated using moderated *t* test method. mRNA levels with p-value < 0.05 and 1.3/-1.3 fold-change or higher/less were stated as significantly changed. Pathway gene enrichment test was performed with the PANTHER Classification System (version 16.0)^47,48^, using Reactome (version 65 Released 2020-11-17) and PANTHER (version 16.0 Released 2020-12-01) annotation datasets. P-values < 0.05 considered as statistically significant.

### Cell proliferation assay

SZ95 cells were collected at 72 h post-transfection by trypsinization, washed in 1 × phosphate buffered saline (PBS), permeabilized, and fixed with 70% cold ethanol for 30 min at 4 °C. Cells were treated with 200 ng/µl RNase (Invitrogen) for 45 min at 37 °C and stained with 5 ng/ml propidium iodide (Invitrogen). Cell cycle phases were assessed with FACSAria flow cytometer (Total cell count: 20.000 for each sample, BD Biosciences, San Jose, CA, USA) and FlowJo software (version X.0.7 BD Biosciences) based on the DNA histogram.

### Lipid content analysis

Intracellular lipid content was measured by Oil Red O staining 72 h after transfection on 96-well plates. Cells were fixed in 10% buffered formaldehyde for 15 min, washed twice with PBS, and stained with Oil Red O (Sigma-Aldrich, 3:2 parts of 0.6% Oil Red O dye dissolved in isopropanol and distilled water) for 15 min. After washed three times with distilled water, Oil red O was washed out with isopropanol, and absorbance was measured with Epoch microplate spectrophotometer (BioTek) at the wavelength of 510 nm.

### Chemotaxis cell migration assay

Monocytes were isolated from whole blood of healthy donors by density centrifugation (Ficoll, Paque Plus, GE Healthcare, Chicago, IL, USA), following separation with CD14 microbeads (Miltenyi Biotech, Bisley, UK), cells were resuspended in RPMI 1640 medium (Invitrogen) supplemented with 1 v/v% L-glutamine (Sigma-Aldrich) and 0,5 v/v% Antibiotic–Antimycotic (penicillin, amphotericin-B, streptomycin, BioSera, Nuaille, France). 1 × 10^5^ isolated monocytes were added to the top of a 5 µm pore cell migration chamber plate (Chemicon QCM 96-well chemotaxis cell migration assay, Temecula, CA, USA). Feeder trays were loaded with supernatants of SZ95 cells transfected with miR-146a-5p mimic, inhibitor or control sequences in triplicates. After 24 h at 37 °C with 5% CO_2_ migrated cells were harvested, lysed and stained with CyQuant GR (Chemicon). Fluorescence was measured with Epoch microplate reader (BioTek) at the wavelength of 520 nm.

### Chromogenic in situ hybridization (CISH)

CISH was performed on 5 µm thick FFPE sections from acne, normal skin specimens and air dried, Carnoy’s-fixed (methanol:acetic acid 3:1, − 20 °C for 5 min) SZ95 sebocyte preparations with 120 nM double-digoxigenin labeled hsa-miR-146a-5p (miRCURY LNA miRNA Detection Probe, HSA-MIR-146A-5P, Cat. No: 339112 YD00619856-BEG, Qiagen), miRNA scramble negative control (miRCURY LNA miRNA Detection Probe, Scramble-miR miRCURY LNA Detec, Cat. No: 339111 YD00699004-BCG, Qiagen), *GNG7* (Custom LNA Detection Probe, Cat. No: 3395005-3DIG, Qiagen) mRNA and mRNA scramble negative control (Scramble-ISH Custom LNA Detection Probe lncRNA and mRNA, Cat. No: 339508 LCD0000002-BDG, Qiagen) locked nucleic acid detection probes according to the manufacturer’s instructions. Sections were treated with 2xproteinase K solution (miRNA ISH Buffer Set, Qiagen) for 15 min, then 120 nM of previously linearized (90 °C for 4 min) probe mix diluted in 1xISH buffer (miRNA ISH Buffer Set, Qiagen) was applied. Hybridization was performed at 54 °C with hsa-miR-146a-5p, 54 °C with *GNG7* and 57 °C with scramble negative control LNA probe for 60 min in a hybridization chamber (StatSpin ThermoBrite, Abbott Molecular, Chicago, IL, USA). For visualization alkaline phosphatase conjugated anti-DIG antibody (Roche) and 4-nitro-blue tetrazolium/5-bromo-4-chloro-indolylphosphate (NBT/BCIP, Roche) AP chromogen substrate was applied for 2 h at 30 °C. Slides were counterstained with liquid-stable nuclear fast red (VWR, Radnor, PA, USA), covered with Eukitt mounting medium (Sigma-Aldrich), coverglassed, and imaged using a Leica DM200LED (Leica, Wetzlar, Germany) microscope.

### Statistical evaluation

Values are presented as mean ± SEM. All experiments have been performed in triplicate. P-values were calculated using Mann–Whitney test, *p < 0.05; **p < 0.01; ***p < 0.001 considered as statistically significant.

## Supplementary Information

Supplementary Information.

## Data Availability

RNA sequencing data have been deposited into the GEO database under accession numbers PRJNA673828 and PRJNA692109. Authors confirm that all relevant data are included in the article and/or its supplementary information files.

## Code availability

RNA sequencing data have been deposited into the GEO database under accession number PRJNA673828 and PRJNA692109.
