# Supplementary material for: miR-146a modulates TLR1/2 and 4 induced inflammation and links it with proliferation and lipid production via the indirect regulation of GNG7 in human SZ95 sebocytes
Source: Sci Rep. 2021 Nov 2;11:21510. doi: 10.1038/s41598-021-00907-1 (PMC8563942; doi:10.1038/s41598-021-00907-1)
Supplement: Supplementary file 1 — Supplementary Information. [file 41598_2021_907_MOESM1_ESM.pdf]

miR-146a modulates TLR1/2 and 4 induced inflammation and links it with proliferation and lipid production via the indirect regulation of GNG7 in human SZ95 sebocytes

Katalin Dull MSc PhD[1], Fruzsina Fazekas MSc[1], Dávid Deák MSc[1], Dóra Kovács MSc PhD[1], Szilárd Póliska MSc PhD[2], Andrea Szegedi MD PhD[1,3], Christos C Zouboulis MD PhD[4], Dániel Törőcsik MD PhD[1]

[1] Department of Dermatology, Faculty of Medicine, University of Debrecen, Debrecen, Hungary

[2] Department of Biochemistry and Molecular Biology, Genomic Medicine and Bioinformatics Core Facility, Faculty of Medicine, University of Debrecen, Debrecen, Hungary

[3] Division of Dermatological Allergology, Department of Dermatology, Faculty of Medicine, University of Debrecen, Debrecen, Hungary

[4] Departments of Dermatology, Venereology, Allergology and Immunology, Dessau Medical Center, Brandenburg Medical School Theodor Fontane and Faculty of Health Sciences Brandenburg, Dessau, Germany

Correspondence: Dániel Törőcsik MD PhD. Department of Dermatology. Faculty of Medicine. University of Debrecen. Debrecen. Hungary; H-4032 Nagyerdei krt. 98.
Telephone: +36 52 255 602
Fax: +36 52 255 736
Email: dtorocsik@gmail.com

Supplementary table 1A. Upregulated miRNAs in TLR1/2 (PAM3CSK4) and TLR4 activated (LPS) SZ95 sebocytes.

FC: fold-change value

LPS 24h

**Accession number**	**p-value**	**FC**
hsa-miR-146a-5p	7.64E-03	2.60
hsa-miR-34a-3p	2.99E-02	2.40
hsa-miR-4517	6.19E-03	2.05
hsa-miR-3613-5p	4.30E-04	1.94
hsa-miR-193b-5p	2.59E-03	1.76
hsa-miR-31-3p	3.43E-02	1.75
hsa-miR-29b-1-5p	3.57E-03	1.71
hsa-miR-582-5p	1.70E-02	1.70
hsa-miR-92a-1-5p	1.81E-02	1.69
hsa-miR-326	7.06E-03	1.67
hsa-miR-22-5p	2.67E-03	1.67
hsa-miR-190a	1.20E-02	1.65
hsa-miR-455-3p	2.14E-02	1.58
hsa-miR-2355-5p	1.10E-05	1.56
hsa-miR-135b-3p	5.98E-03	1.54
hsa-miR-34a-5p	1.59E-02	1.54
hsa-miR-106b-5p	4.45E-05	1.52
hsa-miR-18a-5p	2.39E-02	1.52
hsa-miR-21-3p	4.38E-03	1.51
hsa-miR-323a-3p	3.28E-02	1.51
hsa-miR-16-5p	4.23E-02	1.50
hsa-miR-26a-2-3p	2.80E-04	1.49
hsa-miR-138-5p	3.64E-02	1.49
hsa-miR-99a-5p	1.63E-02	1.45
hsa-miR-147b	1.56E-02	1.45
hsa-miR-27a-3p	5.76E-03	1.45
hsa-miR-4677-3p	2.68E-02	1.45
hsa-miR-651	1.16E-03	1.44
hsa-miR-23a-3p	5.93E-03	1.44
hsa-miR-20a-5p	2.08E-02	1.44
hsa-miR-542-3p	2.80E-02	1.42
hsa-miR-342-3p	8.95E-03	1.41
hsa-miR-19b-3p	3.76E-02	1.40
hsa-miR-200c-3p	3.94E-03	1.40
hsa-miR-339-5p	7.56E-03	1.39
hsa-miR-671-5p	1.02E-02	1.38
hsa-miR-26b-3p	2.55E-02	1.37
hsa-miR-19a-3p	2.71E-02	1.36
hsa-miR-23b-3p	1.53E-02	1.36
hsa-miR-30e-5p	1.03E-02	1.36
hsa-miR-15b-5p	2.03E-02	1.36
hsa-let-7b-5p	8.64E-03	1.35
hsa-miR-487b	3.63E-02	1.35
hsa-miR-548k	1.01E-02	1.34
hsa-miR-589-5p	2.15E-02	1.33
hsa-let-7f-2-3p	2.65E-02	1.33
hsa-miR-193b-3p	3.77E-03	1.32
hsa-miR-29c-3p	3.18E-02	1.31
hsa-miR-126-5p	1.20E-02	1.31
hsa-miR-30a-5p	3.24E-03	1.31
hsa-miR-424-5p	9.34E-03	1.31
hsa-miR-21-5p	2.19E-02	1.31
hsa-miR-98	1.97E-02	1.31
hsa-miR-26b-5p	2.09E-02	1.30

PAM3CSK4 24h

**Accession number**	**p-value**	**FC**
hsa-miR-125b-5p	1.91E-03	2.54
hsa-miR-146a-5p	1.46E-02	2.43
hsa-miR-100-5p	1.55E-02	2.15
hsa-miR-4517	7.48E-04	2.02
hsa-miR-99a-5p	1.89E-03	2.02
hsa-miR-2355-5p	1.47E-02	1.90
hsa-miR-99b-5p	1.11E-02	1.77
hsa-miR-3613-5p	4.54E-03	1.68
hsa-miR-29b-1-5p	4.81E-02	1.67
hsa-miR-33a-3p	1.14E-02	1.64
hsa-miR-147b	4.23E-02	1.62
hsa-miR-22-5p	8.95E-03	1.52
hsa-miR-200a-3p	2.17E-03	1.49
hsa-miR-18a-5p	3.34E-02	1.48
hsa-miR-190a	2.33E-02	1.47
hsa-miR-501-3p	5.49E-03	1.46
hsa-miR-24-3p	2.16E-02	1.46
hsa-miR-106b-5p	1.75E-02	1.45
hsa-miR-135b-5p	4.81E-02	1.43
hsa-miR-23b-3p	4.55E-02	1.41
hsa-miR-16-5p	4.82E-02	1.38
hsa-miR-339-5p	3.12E-02	1.35
hsa-miR-3912	4.07E-02	1.32

Supplementary table 1B. Downregulated miRNAs in TLR1/2 (PAM3CSK4) and TLR4 activated (LPS) SZ95 sebocytes.

FC: fold-change value

LPS 24h

Accession number	p-value	FC
hsa-miR-486-3p	1.69E-02	-1.33
hsa-miR-3065-5p	1.06E-02	-1.36
hsa-miR-454-5p	3.05E-02	-1.44

PAM3CSK4 24h

Accession number	p-value	FC
hsa-miR-532-3p	3.36E-02	-1.33
hsa-miR-1304-3p	4.67E-02	-1.35
hsa-miR-3605-3p	2.78E-02	-1.77
hsa-miR-628-3p	3.82E-02	-1.78

Supplementary table 2A. Significantly upregulated mRNAs in miR-146a inhibitor treated SZ95 sebocytes (72 hours)

FC: fold-change value

Gene symbol	p-value	FC
CLEC7A	4.46E-03	5.26
C21orf88	4.32E-03	4.86
SUMO4	2.72E-03	4.46
GNG7	8.02E-03	3.98
LRRTM2	4.29E-05	3.82
COL1A2	2.89E-03	3.81
SLC6A14	5.89E-04	3.47
SPINK1	3.74E-03	3.41
LMO3	1.02E-03	3.35
RNF157-AS1	3.13E-02	3.09
AC135178.1	4.62E-02	3.04
FOXF1	1.07E-02	2.96
MCF2L	1.77E-02	2.84
COL4A3	1.17E-02	2.83
LINC00551	7.14E-03	2.78
NCALD	9.92E-03	2.74
PIGR	3.77E-02	2.61
SULT1E1	6.61E-03	2.60
KIAA1024	2.36E-02	2.58
DUSP13	4.66E-03	2.53
CD177	2.04E-02	2.52
COLEC10	2.98E-02	2.50
C4BPB	6.50E-03	2.46
PSG2	1.06E-02	2.45
ARL14EPL	3.94E-02	2.42
PDE7B	7.31E-03	2.41
PPP1R32	4.13E-04	2.39
MYO1H	2.57E-02	2.36
RSF1-IT2	2.53E-02	2.31
AL355490.1	4.08E-02	2.30
C11orf16	1.25E-02	2.28
ARID4B-IT1	1.44E-02	2.23
NIM1	3.43E-03	2.23
BIRC7	1.62E-02	2.21
SAA4	4.82E-02	2.21
FRMPD2	4.35E-02	2.20
CD207	4.04E-02	2.17
OR51B2	1.13E-02	2.15
SEMA6D	4.59E-02	2.14
MLANA	2.12E-02	2.12
C1QTNF5	7.71E-04	2.10
MFRP	7.71E-04	2.10
ANG	1.05E-02	2.10
TNFRSF8	2.93E-02	2.09
GLIPR1L1	4.06E-02	2.09
SCARNA13	1.81E-02	2.06
UBOX5-AS1	2.19E-02	2.06
LRRC10B	3.54E-02	2.05
FRRS1L	3.13E-02	2.05
FRMPD3	2.62E-02	1.99
OXCT1-AS1	4.51E-02	1.99
LINC00035	2.47E-02	1.97
CLDN16	2.35E-02	1.92
RASD2	3.11E-02	1.92
CD37	6.18E-03	1.91
CYP4B1	5.69E-04	1.91
RP11-404P21.8	4.90E-02	1.90
SIRT4	1.40E-02	1.89
ACKR4	8.52E-03	1.89
PDE6A	3.43E-02	1.88
AKR1C1	4.39E-02	1.87
GPR114	1.34E-02	1.86
SERPINA3	4.35E-02	1.84
CCDC146	3.47E-02	1.80
AC002365.1	2.80E-02	1.80
LRRC69	3.16E-02	1.79
ACSL6	9.49E-04	1.78
ANGPTL2	6.53E-03	1.78
UGT1A6	4.28E-02	1.78
NWD1	3.98E-02	1.77
PLEKHD1	4.93E-02	1.77
WDR88	2.89E-03	1.77
LRRC19	4.49E-02	1.76
BTBD8	3.84E-02	1.76
SHE	4.94E-02	1.76
PPIAL4C	6.69E-04	1.74
CALHM3	3.93E-02	1.74
PLLP	1.04E-02	1.73
STAC2	1.30E-02	1.73
ALPPL2	4.95E-02	1.72
SLCO4C1	9.24E-03	1.71
INSL4	4.34E-02	1.70
SEMA5A	1.40E-02	1.70
WDR72	4.53E-04	1.69
PRUNE2	4.27E-02	1.67
DHRS9	4.88E-02	1.66
NEBL	2.72E-02	1.66
MAPK10	3.60E-02	1.66
IGDCC4	4.14E-02	1.65
CAPN11	4.30E-03	1.65
CDKL5	4.04E-02	1.64
ERI3-IT1	4.89E-02	1.63
TOMM20L	1.49E-02	1.63
BNIPL	4.56E-03	1.63
CST5	3.90E-02	1.62
PADI2	4.95E-02	1.60
LINGO2	2.01E-02	1.60
C17orf72	2.36E-02	1.60
TFF2	2.55E-02	1.58
TMEM45B	1.13E-02	1.58
SNORD69	1.80E-02	1.57
MIR7-3HG	5.00E-02	1.57

Gene symbol	p-value	FC
GRTP1-AS1	2.83E-02	1.56
RAB9B	2.99E-03	1.56
ANKRD24	4.50E-03	1.56
AQP3	6.97E-03	1.55
EQTN	4.12E-02	1.55
MAMDC2	4.88E-02	1.55
DAPK1	2.57E-02	1.55
RARB	2.12E-02	1.54
PADI1	4.00E-02	1.54
PPIAL4A	2.08E-03	1.52
FXYD6	3.71E-02	1.51
KL	3.88E-02	1.51
AP006621.5	3.32E-02	1.51
ANK1	2.46E-02	1.50
GOLGA8N	1.07E-02	1.50
UNC5B	5.49E-03	1.49
C6orf223	1.71E-02	1.49
ADORA1	4.82E-02	1.48
NAALADL2	2.91E-02	1.48
CLIC5	4.80E-02	1.47
PTPRR	1.88E-02	1.46
TCP11L2	2.20E-02	1.46
FOXN4	3.95E-02	1.46
ESRG	4.99E-02	1.45
MEOX1	4.63E-02	1.45
HMBOX1	4.37E-02	1.44
NBEA	3.93E-02	1.44
SATB1	3.94E-02	1.43
KIAA1211	2.84E-02	1.43
NRG4	2.59E-02	1.42
ANXA9	3.35E-02	1.41
TMEM51-AS1	1.56E-02	1.41
RIC3	2.54E-02	1.40
STEAP4	3.60E-02	1.39
PLCE1	1.83E-02	1.39
ST8SIA4	3.16E-02	1.39
SYNE1	4.59E-02	1.39
ABO	1.72E-02	1.36
SCAPER	3.82E-02	1.36
SDR16C5	4.59E-02	1.36
MIR31HG	1.55E-02	1.35
DDX11L2	4.17E-02	1.34
INPP5D	3.01E-02	1.34
Y_RNA	3.76E-02	1.34
KIAA1217	3.10E-02	1.34
HECA	3.75E-02	1.34
NMNAT2	4.20E-02	1.33
MYO7A	2.99E-02	1.33
TRIM45	3.32E-02	1.33
ATP7A	3.01E-02	1.32
C22orf23	4.85E-02	1.32
BTN3A3	4.42E-02	1.32
ATP2B4	3.17E-02	1.31
PDE5A	4.59E-02	1.31
RAPGEFL1	4.72E-02	1.30

Supplementary table 2B. Significantly downregulated mRNAs in miR-146a inhibitor treated SZ95 sebocytes (72 hours)

FC: fold-change value

Gene symbol	p-value	FC
ZNF527	4.79E-02	-1.29
WDR49	2.13E-02	-1.32
LRP5L	4.87E-02	-1.33
SERPINB2	4.50E-02	-1.33
PTCD1	4.07E-02	-1.33
HIST2H2AA4	3.14E-02	-1.33
ISL2	2.69E-02	-1.34
HIST2H2AA3	3.95E-02	-1.34
AP4S1	2.01E-02	-1.37
PHLDA2	4.18E-02	-1.38
SIX2	2.26E-02	-1.39
ZNF653	3.69E-02	-1.40
SERTAD1	3.06E-02	-1.41
LENG9	4.29E-02	-1.41
AC004466.1	4.35E-02	-1.42
PRR9	2.44E-02	-1.43
HIST2H4B	1.09E-02	-1.44
SHOX2	2.08E-02	-1.45
SLC34A3	6.72E-03	-1.45
HIST2H4A	1.07E-02	-1.45
RP13-996F3.4	1.48E-02	-1.47
AC003002.4	2.81E-02	-1.47
HAS3	4.15E-02	-1.47
ACRC	1.87E-02	-1.47
TMEM200A	1.22E-02	-1.48
MIR17HG	6.55E-03	-1.48
KCNMA1	3.77E-02	-1.49
CCDC85A	2.08E-02	-1.49
ANKRD16	2.22E-02	-1.50
TRBJ2-7	4.81E-02	-1.51
TWIST1	3.27E-03	-1.52
MIR663A	2.06E-02	-1.53
FAM183A	1.96E-02	-1.53
ACTL10	2.71E-02	-1.53
KLHL35	4.88E-02	-1.54
P2RY1	2.64E-02	-1.55
U3	1.56E-02	-1.57
SERTAD4-AS1	9.79E-03	-1.58
HBA1	3.91E-02	-1.59
VAC14-AS1	1.03E-02	-1.60
TBR1	1.79E-03	-1.61
G0S2	5.73E-03	-1.61
CXCL1	4.64E-02	-1.64
FERMT3	2.68E-02	-1.64
GPT	7.46E-03	-1.65
AC006014.1	2.47E-02	-1.66
FJX1	8.73E-03	-1.67
PCP4L1	2.34E-02	-1.67
LINC00176	2.59E-02	-1.67
IL23A	1.26E-02	-1.68
RADIL	4.47E-04	-1.69

Gene symbol	p-value	FC
VGF	9.21E-03	-1.69
EGR2	3.90E-02	-1.70
7SK	4.85E-03	-1.71
IL18BP	1.76E-02	-1.73
CTD-2510F5.6	2.03E-03	-1.73
ADAMTS9	3.34E-04	-1.73
CNPY2	1.37E-02	-1.74
GAS6-AS1	4.11E-02	-1.75
FZD8	2.78E-03	-1.75
TDO2	1.05E-03	-1.76
SPRR3	3.21E-02	-1.77
TBX15	1.09E-03	-1.77
FZD9	2.86E-02	-1.78
QRICH2	2.40E-02	-1.78
MPL	3.57E-02	-1.78
PCDHAC1	1.26E-02	-1.80
SNORD78	1.87E-03	-1.80
HIST1H2BN	3.60E-02	-1.81
C19orf80	4.35E-02	-1.85
NOSTRIN	6.29E-03	-1.86
GGTLC2	6.52E-03	-1.87
HIST1H2BH	1.76E-02	-1.88
LINC00562	5.00E-02	-1.88
PTPRZ1	2.21E-02	-1.91
ARHGAP22	3.99E-05	-1.92
INSRR	3.75E-02	-1.93
ZCCHC18	2.22E-02	-1.97
HAPLN2	2.39E-02	-1.98
SNORD46	1.30E-02	-2.00
CCL26	5.88E-04	-2.00
CPNE4	2.89E-02	-2.01
PPP1R1C	1.17E-02	-2.03
FOXP1-IT1	1.76E-02	-2.03
MUC6	1.17E-02	-2.03
FGF1	8.77E-03	-2.04
SPINK5	3.17E-02	-2.04
AKR7L	3.34E-02	-2.05
FGF18	3.99E-02	-2.06
PRKD1	4.29E-02	-2.07
AC025287.1	5.74E-04	-2.08
EBF4	3.93E-02	-2.10
PDZD9	2.82E-02	-2.11
FAAH2	3.97E-02	-2.13
AC006435.1	3.86E-02	-2.18
HIST1H2BO	2.08E-02	-2.20
ANXA13	1.21E-02	-2.22
OVOL3	3.47E-02	-2.26
FLT4	1.34E-02	-2.27
SNORD67	4.73E-02	-2.28
USP17L2	3.83E-03	-2.29
CTSW	2.39E-02	-2.32

Gene symbol	p-value	FC
GRASP	1.69E-02	-2.34
FAM153A	4.77E-02	-2.34
GJA1	2.88E-02	-2.36
SLA2	7.48E-03	-2.38
PCDH10	2.39E-02	-2.41
NKX2-5	4.08E-02	-2.41
IL18RAP	4.35E-03	-2.42
SNORA3	4.97E-02	-2.43
FCGR1A	7.66E-03	-2.44
ZNRF3-AS1	1.57E-02	-2.47
MADCAM1	4.65E-02	-2.48
LHX5	3.06E-02	-2.50
SHROOM4	9.66E-04	-2.50
NOX1	6.42E-03	-2.53
ETNPPL	3.16E-02	-2.54
HAVCR2	1.27E-05	-2.57
SLC36A2	8.46E-03	-2.64
RUNDC3B	2.84E-02	-2.68
AXDND1	3.61E-02	-2.69
DNAJB13	1.01E-02	-2.70
GRM8	4.92E-02	-2.74
TTC40	2.37E-02	-2.79
C20orf26	4.98E-02	-2.80
CACNA1B	7.03E-03	-2.81
LRRC66	3.86E-02	-2.83
SULT1B1	6.99E-03	-2.91
JAM2	4.81E-03	-3.00
AMN	2.98E-03	-3.03
CYP2E1	8.47E-03	-3.03
PNLIPRP2	2.00E-02	-3.06
CRHBP	1.18E-05	-3.16
CCDC144NL	1.81E-02	-3.21
DNALI1	1.36E-02	-3.26
MIR3614	4.74E-02	-3.27
LHX3	3.39E-02	-3.61
ZNF80	6.95E-03	-3.61
ABCB6	2.22E-04	-3.68
LPPR5	3.30E-04	-3.69
PAX5	4.40E-03	-3.76
LDLRAD2	1.52E-03	-3.76
SLC24A3	1.06E-02	-3.79
SNORD23	3.96E-04	-4.58
MTOR-AS1	1.00E-04	-10.88
